# The Scientific Investigations of the Bureau of Animal Industry

**Published:** 1892-11

**Authors:** D. E. Salmon


### THE SCIENTIFIC INVESTIGATIONS OF THE BUREAU OF ANIMAL INDUSTRY.*

BY DR. D. E. SALMON.

Considered as an abstract proposition there is probably no member of the veterinary profession who is not willing to admit the desirability of original scientific research. To the liberal-minded members of the profession, the value of such research for the development of science, for the perfection of our practice and for raising our profession to a higher plane in the eyes of the world must be apparent. It has been generally agreed that the reproach of both the medical and veterinary professions of this country has been the lack of activity and the poverty of results in the field of scientific investigation.

* Paper read before the 29th annual meeting of the United States Veterinary Medical Association.

Every veterinarian who takes pride in his profession and is at the same time a patriotic citizen of the United States, must have a feeling of regret that such a condition of affairs could exist, and he must equally feel an interest in all efforts that are made to correct it. While we should feel no jealousy of the scientific workers of the old world and should welcome every contribution which they make to the fund of existing knowledge, we should certainly rejoice at every effort made in a scientific spirit in this country to conduct researches of the same quality and the same value as are made by the most advanced students in other lands.

This, it appears to me, is a legitimate deduction which may be drawn without fear from our common knowledge of patriotism and professional pride in all times and all countries. More than this, if I mistake not, it is one of the objects for which this Association exists to promote and encourage scientific research. If I am correct in this, it follows that both the young experimenter who has taken his first steps in original research, and the more experienced one whose ambition leads him to attack the most difficult problems, should equally meet with welcome and favor when they bring their trophies for the adornment of the organization of which they are members and which represents the veterinary profession of the country.

In saying this it is not my purpose to take the ground that a paper read before this Association, or the results of the investigations of members published elsewhere, should not be discussed and criticised. On the contrary, it may be freely admitted that it is one of the most important of our privileges to discuss such papers, and in so doing to bring out points which may have been obscure or to call attention to conclusions which may be untenable. But the investigator has a right to expect in such discussions that this Association is gratified at every effort, however humble it may be, to pierce the mysteries of nature ; and that when he brings his results before it, or when these are introduced by others, he will be received with courtesy, and that any criticisms will be fair and truthful.

It is vital to the success of this Association that such expectations should not meet with disappointment. Our great need is that we should have more original investigations—that the many peculiar problems which confront us in this country should be solved, and that they should be solved, if possible, by our own members.

These remarks are made by way of introduction to my paper because this is the first occasion, since I have been a member of this organization, that it has been necessary for me to defend my work and my character from unjust assaults, made, not in the heat of debate by an individual, but with deliberation by one of our most important committees, and at a meeting from which I was unavoidably absent. The chief points of that report which relate to me I have already discussed in an open letter which you have all had an opportunity to read. (Jour. of Comp. Med. and Vet. Arch. and Am'n. Vet. Review, both for January, 1892). In that, however, I was unable for want of space to treat with any detail the scientific questions involved, and consequently I shall take some of these up at this time.

To remind you of the tone of the report made at the last meeting of this Association, I will quote only two sentences, which are taken from near the end; they read as follows:

"Having taken the investigation of swine diseases as a fair sample of this Bureau's scientific labors, are we to be expected to place any dependence upon the accuracy of the statements emanating from its officers concerning such work, especially when they conflict with the results obtained by men like Paquin and Billings, unless the work of the former is confirmed by experimeuts conducted by independent and unprejudiced observers of recognized ability?

"How can we, as a profession, feel anything but disgraced when we think of the opinions which must be held in Koch's laboratory, the greatest bacteriological laboratory in the world, concerning our Bureau of Animal Industry, and its scientific work?"

Is there any one here who can find in those two questions any favorable appreciation of the scientific investigations of the Bureau of Animal Industry, or any encouragement for the scientists of that Bureau, several of whom are members of this Association, to continue their labors in this, their chosen field? Quite the contrary. The entire field of scientific investigation in this country can be explored without finding an example of such rank injustice or of such uncalled-for defamation. It is for this reason that I propose to take up the matter at this time and show how accurate have been the reports of the Bureau and how untenable the positions of those who assailed them.

Billings has published an abstract of the paper of Frosch, referred to so much in Peters' report, under the title of "Billings Investigations Vindicated," and Peters' endeavors to give the impression that Frosch's report endorses the various positions which

Billings has assumed, and shows that the Bureau reports have been incorrect and unreliable. It is true that he does not undertake to prove this by a citation of the facts, a course which would no doubt have been inconveniently troublesome, but his whole argument gives this impression, as is sufficiently shown by the short quotation which I have made.

Now, what are the facts about this subject? In the first report of the Bureau, that containing the work of 1884, the cause of swine plague was spoken of and figured in liquid cultures as a dumb-bell microcroccus. In recent years I have laid no stress on that early work because it was done soon after I went to Washington when bacteriology in this country was in its infancy, and when the Bureau had neither the instruments, the laboratory facilities, nor the assistance which we now know to be essential for such investigations. It is also well known that I had many other duties at that time, such as the organization of the Bureau and the investigation and control of an outbreak of pleuro-pneumonia in the Western States which caused several long absences from Washington, one of which was of more than three months' duration. This is no reason why my scientific conclusions at that period should be received on any less evidence than is required from other people, but it is an explanation as to why the germ then figured was not studied under different conditions of culture and in its effects upon different animals so that its identification by other investigators would be easy and certain.

It is evident, of course, in the light of to-day, that we cannot claim to have established the fact that the micrococcus of 1884 was a specific pathogenic germ, because we did not give the means for the subsequent identification of this germ either by other investigators or ourselves. It is significant, however, that a disease of swine has since been studied in Germany and in the United States, the bacteria of which grow in liquid cultures as did the germ figured in the report of 1884. There is only one discrepancy, and that was explained long ago and before any one else has discovered it. It was stated in that report that the micrococcus liquified gelatine. This error was owing to my cultures being then made in liquid media after the Pasteur method and laboratory facilities, as on account of lack of assistance, the gelatine method was impracticable. Because of changes in the laboratory and my long absence, my cultures became shifted about and in some cases the labels misplaced. On my return it became necessary to hurriedly prepare my report and a culture supposed to be the same as that previously photographed was plated to determine its effect on gelatine. The gelatine on this plate was liquefied, and on the strength of this one experiment the statement above referred to was made in the report. The error was afterwards discovered and corrected by me, but it is still referred to by unscrupulous writers as a reason why the micrococcus of 1884 could not be identical with the micrococcus described in 1886 and in subsequent reports.

A discussion of this question is of little importance at the present day, particularly when the one who made the investigations in question has since had the whole field worked over with all the appliances of modern science, and has unreservedly published the results. These old reports which were made before exact bacteriological methods were introduced into this country, are only referred to now by those who wish to discredit the work of the Bureau ; but they forget that if it is impossible at this time to prove the conclusion that the micrococcus of 1884 was identical with that of 1886, it is equally impossible for them to prove that the two were not identical.

What is well known to all of these writers is that under my direction the laboratory facilities of the Bureau were rapidly increased, that the work of experimentation was divided, that proper instruments were obtained, and that the results from that time to the present have been equal in accuracy, detail and importance with those obtained in any other laboratory in the world during the same period. In the report for 1885 the microbe of hog cholera was accurately described, and you may ransack the literature of the world for any description made previous to that time by which you can identify that germ.

In the report for 1886 the germ of the American swine plague was described, and the differences between this and the hog cholera germ were plainly indicated. In the report for 1885 the disease investigated was referred to as swine plague, but before the following report appeared we received the report of Schütz on *Schweineseuche* and also found that we had a disease of the same nature in the United States. With the two diseases before us we decided to call the one swine plague which appeared to be identical with the *Schweineseuche* of Germany. Since *Schweineseuche* can only be translated as *swine plague*, this course appeared to be necessary to avoid confusion ; and if this example had been followed by others who wrote afterwards, the confusion which is now found in the nomenclature would not exist.

The impression which Billings and Peters attempt to give, in that the investigations of Frosch sustain the claims of Billings and refute the conclusions of the Bureau of Animal Industry. This impression is absolutely false, as can be easily proved. It is true that Frosch was led, by reading Billings' misleading, and in many respects incorrect reports, to express opinions uncomplimentary to the Bureau and discreditable to himself on subjects of which he knew no more than any other reader of the reports; but so far as his scientific investigations go, and that is all the evidence offered by him which is worth anything, he sustains the reports of the Bureau in every important respect.

After what has been read in your presence and published by our journals, I suppose the most of you are surprised that I can make such a statement. If I make it I am prepared to prove it, and I feel sure that no man can contest the evidence which I offer.

What was the first position taken by Billings in regard to the germ which was described by the Bureau in 1885? We will take his report published in 1888 as authority, as he then had given two years to the investigation, and he certainly cannot, therefore, accuse us of using his prematurely expressed opinions in making this argument. After two years of study and investigation, with the advantage of having our reports for 1885 and 1886 in his possession, he deliberately states that no such germ as our hog cholera germ was found by him; that such a germ has no existence, and that our germ was a fabrication, or, to use his own words, "a forgery."

Referring to the bureau experiments of 1885 he says: "Furthermore, their value is absolutely nullified, for the simple reason that no snch germ exists in connection with swine plague as that described above." (University of Nebraska; Second Report from the Patho-Biological Laboratory, 1888, p. 54.)

Again he says: "By thus ignoring them, he silently admits that they were not 'un,' but 'well-founded,' statements, and hence, as he has not, and cannot, prove them to have been, or be. erroneous, and those which he alludes to of 1885 are of the same nature, I emphatically declare that all Salmon's assertions with regard to the specific microscopical appearances of any true and specific pathogenic micro-organism in connection with hog cholera to be one continued series of 'unfounded statements' down to the issue of his report of 1886." (Loc. cit., p. 68.)

Once more he says: "I positively assert that Salmon's assertion of a distinct germ for the disease which he now calls hog cholera is erroneous, and that the description of that object is forgery; that it does not exist or occur in any form of the American swine plague, and that neither Salmon nor any one else can demonstrate the presence of that object in the tissues or blood of any hog that has died of swine plague in any part of this country, if the examination is made before cadaveric changes have taken place.

"That the object described by Salmon as the germ of the hog cholera cannot be cultivated from the tissues of any animal that has died of hog cholera or swine plague." (l. c., p. 74.)

He returns to this subject as follows: "That this substance does not represent a spore condition or have any relation to spores, is, to my mind, entirely beyond all question, as I have searched most diligently for spores in old and fresh cultures, and others made at all kinds of temperatures within the biological limits of these organisms, my search being inspired by the description of what I pronounce a forgery of a germ (which represents a spore), as the cause of swine plague, by Mr. Salmon in 1885, and again in 1886, as the cause of an assumed porcine pest, to which Mr. Salmon now gives the name of 'hog cholera.' This Salmon object does not exist, never has existed, and never will have any etiological connection with the American swine plague, as has been conclusively demonstrated in this report." (l. c., p. 111.)

In his *post mortem* notes he says: "Though the lesions here described must and will completely fill Mr. Salmon's picture of his 'hog cholera,' described in his report of 1886, but in this, as in every other case, Mr. Salmon's specific 'hog cholera microbe' was missed, and 'it ever will be missed' in the American swine plague, no matter who seeks it or how much time they spend in the hunt." (l. c., p. 136.)

In another *post mortem* note he says, parenthetically: "It does not seem as if any one could have seen more extensive lesions in the large intestine, which Mr. Salmon says are characteristic of his 'hog cholera,' yet his manufactured germ of 'hog cholera' never had any connection with the lesions in this hog." (l. c., p. 163.)

At the conclusion of his *post mortem* notes he adds: "After several hours spent at this useless job, we were unable to find any other micro-organism than that invariably found in every case of swine plague we have investigated, and which has been described as a 'belted ovoid organism which colors at its pole ends," and which bears no resemblance to the Washingtonian bureaucratic nondescript." (l. c., p. 164.)

I have already made enough quotations to demonstrate Billings' position on this question in 1888, when his report was written; but it is my object to show that this was the main idea of that report—the conclusion upon which the whole structure was built. As so much depends upon this one fact I trust that you will bear with me while I demonstrate that from the beginning to the end of the four hundred pages these assertions are repeated as the corner-stone of the whole argument.

Describing an autopsy made by himself and Dr. Roberts, of Creighton, Neb., he adds:

"The above case is of special interest and is only introduced here because if there ever was a case of swine plague which absolutely corresponded in every particular to Mr. Salmon's 'hog cholera,' this one did. Hence the most exact search was made in seeking micro-organisms, but in the blood, in the spleen, in the liver, in the lymph-glands, both Dr. Roberts and myself could not find any other organism than that described by me in this report as the only germ of swine plague. Cultures were made and the same organism developed. Dr. Roberts being here with me and interested in this work, as well as being a regent of the University, he was doubly interested in endeavoring to find anything like the organism described by Mr. Salmon as occurring, according to him, in just such cases. Here was no room to say with Salmon, the lungs being affected, the germ of swine plague and that of hog cholera may both be present. After spending more than an hour in examining and re-examining the fresh organs, all of which were kept carefully covered, except for the moment necessary to clip out a small piece of tissue, we gave up the search, finding nothing but the one organism, and nothing resembling in any way the one described by Mr. Salmon in 1885 as the cause of swine plague, which had no polar staining whatever, and in 1886 as the cause of his 'hog cholera.'"

"Such testimony as this should be conclusive. A mistake is absolutely impossible under the circumstances. This result simply corresponds with over 500 such experiences." (l. c. p. 259.)

Finally, referring to my experiments in producing immunity in pigeons with the chemical products developed during the growth of the hog cholera bacterium, he writes:

"Hueppe seems to be absolutely unaware of the fact that the experiments he refers to were published in Mr. Salmon's report of 1885, when he said that the peculiar organism, which he then called the 'new microbe of swine plague,' was entirely different from that of Schütz. Hueppe is also unaware of the fact that that same organism suddenly became the cause of Mr. Salmon's 'hog cholera' in 1886, as he is that that same organism has no existence in the American swine plague." (l. c. p. 394.)

Two pages farther on he says:

"With this apparently positive evidence in his hands, why did not Mr. Salmon proceed with such valuable work in 1886 aud 1887, not to speak of the present year?

"Why does he not mention any further experiments of the same kind in his report of 1886, or his later publications?

"What is he employed for but to do this thing?

"The true answer is easy to discover!

"*Because Mr. Salmon is fully aware that no such organism as his swine plague 'new microbe' of 1885, or his 'hog cholera' of 1886, '87 and '88, exists as an etiological moment in swine plague.*" (l. c. p. 396.)

These extracts are certainly sufficient to demonstrate Billings' conclusion as to the germ of hog cholera up to the time his report was written in 1888.

Was he right or wrong in his conclusion that no such germ as the hog cholera microbe existed, and that it had never been found and never would be found in the American swine disease? Is there any one in this Association, or indeed, any one in the country, who believes that Billings was right and the Bureau wrong on this fundamental question? You all know better. You know that all who have since investigated the question have acknowledged the reality of the hog cholera microbe and its etiological relation to that disease. Even Billings himself has since adopted it, and in the September (1892) number of the Journal of Comparative Medicine he gives a figure of it, which does not correspond in the least with the figures in his report or with his description of it as an "ovoid, belted germ," morphologically identical with the microbe of *Schweineseuche*.

All the statements of his which I have quoted were, therefore, radically wrong so far as they denied the existence and the etiological relation of the hog cholera germ. Were they wrong as to the germ which he found at that time? This question we will consider later, but it should be remarked here that Billings claims to be a bacteriologist and that consequently he should be able to discriminate between two such radically different germs, especially after the points of difference have been so carefully pointed out as they were in the Report of the Bureau of 1886.

Under such circumstances the investigator's own conclusion must be taken, when it is given in such positive language, and no one has a right to go behind it and claim that the investigator meant something different from what he wrote. The investigator has the germs under his own eyes, and if he cannot tell what germ he has, how ridiculous it would be for some one else, who has never seen the germ, to attempt to decide from the written description of the man who himself cannot tell.

In the face of these facts, how could the Committee on Intelligence and Education pretend in their report at the Washington meeting that the discovery of the identity of the Billings swine plague germ (as now accepted by him) with the hog cholera germ of the Bureau, sustained the claims of Billings and showed the Bureau reports to be unreliable? How could the committee record its belief that his work was "really correct and valuable," and that ours was a disgrace to the profession? How absurd this is when it is apparent that the man, who in his report reiterated again and again his conclusion that no such germ existed, is only too glad to give the impression now that this was the germ he was working with from the first

How could the committee with such undisguised exultation tell us that: "Dr. Billings boldly announces that he found his germ of swine plague in July, 1886, among the first pigs that he examined in Nebraska, which had died of the disease." Did the committee stop to inquire what germ he found at that time? Did they take into account his many denials of the existence of any germ which had the least resemblance to the hog cholera germ of the Bureau reports? If so, how can they come before this meeting and try to give the impression that the germ which Billings first found in Nebraska was identical with the hog cholera germ of the Bureau? These are serious questions, and relating, as they do, to an attack upon the reputation of individual members of this Association, as well as upon that of a Bureau in which veterinarians are especially interested, we have a right to demand that they be answered.

We may also ask with much reason how Frosch can consider that Billings' inoculations of hogs with a germ which the experimenter insists was not the hog cholera germ and has no resemblance to it, nevertheless fill out the gaps and bridge over the doubts which he believes to exist on account of what he calls our objectionable methods in proving the pathogenic action of the hog cholera germ? He certainly has no right to assume that because Billings sent him the hog cholera germ as his swine plague germ, in 1889, that this was this germ with which he worked previous to 1888, when Billings expressly says so often in his report that it was not that germ. This is one of Frosch's conclusions drawn from data other than that obtained from his bacteriological investigations, and it shows how utterly unreliable he is when he leaves his own results and attempts to analyze and weigh reports of investigations written in the English language. For no one can suppose that inoculations with one germ would be of any value towards proving the pathogenic action of an entirely different organism.

This is only one of the attempts which he has made to criticise the Bureau and endorse the work of Billings, in cases where an examination of the latter's report does not sustain his language. For instance, he criticises the Bureau reports because the cultures used for inoculation were not derived from single colonies on gelatine plates, and yet he unhesitatingly accepts the inoculations of Billings as bridging over this gap, although we search in vain through Billings' report for any evidence that his inoculation material was obtained from plate cultures. It should also be remembered that it is very evident from the communication of Schütz on the *Schweineseuche* of Germany which appeared about the same time as our report for 1885, that plate cultures were not used for obtaining the virus with which his inoculations were made. And yet his conclusions have not been contested either by Frosch or others. When his attention was called to this by Dr. Smith, Frosch contented himself by characterizing the allusion to Schütz as "wholly unjustifiable" without troubling himself to go into particulars. As the cases were in every respect parallel, the unbiassed reader must continue to wonder what subtle distinction was conjured up by the fertile imagination of the German investigator.

"Again," Frosch says in reply to Smith, "after the determination of the identity with Salmon's hog cholera bacterium, I was compelled, in testing the chief question of the pathogenic nature of this bacterium for pigs, for which experiments of my own were not possible, to base myself upon the statements of F. Billings. For this Mr. Smith must blame himself, since—I regret to have to repeat it—the methods and experiments laid down in the reports of the Bureau of Animal Industry for the years 1885 to 1888 did not in a desirable manner come up to the standards demanded in the determination of a new infectious disease. I need not make myself guilty of a repetition of that which has been said in my article, since Mr. Smith agrees with me in this regard with reference to the report of 1885. On the other hand, even a superficial examination of the other mentioned reports shows that Koch's methods were not applied exclusively or exactly, although this was unconditionally demanded by the creation of a second infectious agent of swine plague which appears at the same time and is so closely related to it."

The query which naturally suggests itself here is, if an exact and exclusive application of Koch's method was required to make the Bureau investigations acceptable to Frosch, why was not a similar application of Koch's method necessary to make Billings' investigations acceptable to him? He makes two objections to our inoculation experiments; first that the virus was not obtained from plate cultures, and second, that the control animals in some cases died as soon, or even sooner, than the inoculated ones. This last objection should carry but little weight, because, as pointed out by Smith, an inspection of the records shows in every case where the controls died at the same time or earlier than the experimental animals, that this was accounted for by the disease being of a more acute type.

When we analyze the report of Billings we find that what Frosch calls extensive and valuable experiments consists in thirty-three pigs, all told, which were inoculated, and two which were fed. Of the thirty-three inoculated at different times, it is stated that two died, that three were killed for examination, and that "the same general results occurred" with six others as with one of those that were killed. The two that were fed died. There is not a particle of evidence that the virus used in any of these cases was obtained from plate cultures, and control animals did not complicate results for the very good reason that there were none in any of the experiments.

It surely is not necessary for me to dwell at greater length upon Frosch's bias in Billings' favor, and the unaccountable conclusions to which this led him. But these are minor points which should not lead us to undervalue his contribution to this subject, when considered as a whole. The serious thing to us is that it was these details in his statements—details based upon the flimsiest foundation, which attracted the attention of the Committee on Intelligence and Education, blinding the members to the real facts of value which were brought out by Frosch, and leading them to denounce in unmeasured terms the investigations of the Bureau of Animal Industry and all individuals connected with them.

It may be remarked here that no one can steal a discovery from another by objecting to the methods by which that discovery was made. Koch's early investigations of the anthrax bacillus were made by methods which would certainly be considered very objectionable from the standpoint of to-day, but his demonstration of the etiological relation of the bacillus to the disease cannot be taken from him on that account. And so when a germ is isolated and described as carefully as was the hog cholera bacillus by the Bureau of Animal Industry, when its effects on various animals and its biologic characters are made known, the investigators ought not and cannot be cheated out of due credit when their conclusions are confirmed by competent scientists. The value of investigations is shown by their fruits, and if the conclusions are proved to be correct, the methods may in the end turn out to be as satisfactory as those used by that other class of investigators whose chief object in life is to assail and embarrass the workers who are building up science and advancing the interests of their profession.

We will now turn to Frosch's report and see what there is of importance which sustains or conflicts with the reports of the Bureau. His most important conclusion is that "the bacterium of Salmon's hog cholera and that of Billings' swine plague are identical, and that the same is to be looked upon as the cause of the American swine plague." That is to say, the bacterium sent to Koch by Billings in 1889 was identical with the hog cholera bacterium described in the bureau reports for 1885 and 1886.

There was nothing unexpected in this conclusion, for we already knew from the report of the Commission of Inquiry appointed by the Department of Agriculture, and from the investigations of Professor Welch, of Johns Hopkins University, that Billings at this period had adopted our hog cholera germ as the cause of his swine plague. The surprise came when Billings placed our hog cholera germ into the hands of the commission as the cause of his swine plague, after he had so long denied the existence of that germ.

If we assume, for the present, notwithstanding the denials in his report, that this writer had been working with the hog cholera germ during the first two years of his investigations, that is, from July, 1886, to July, 1888, then it becomes a point of great interest to learn whether Frosch was able to identify the germ from our description. If he could not, then there might be some excuse for Billings' frequent denials of its existence. It should be remembered that the Commission of Inquiry and Professor Welch had both expressed their conclusion that our description was correct and sufficient for the identification of the germ. Now, what does Frosch say?

He says: "We will hence briefly review Salmon's statements bearing in this direction, in order to prove how far the same correspond, free from objection with the criteria set up by Koch for an infection cause.

"If the reports of Salmon concerning the hog cholera bacterium are next viewed from this standpoint, then a sufficient characterization, morphologic as well as biologic, of the same is given which allows no doubt to arise concerning its identity."

Again he writes: "A culture of this hog cholera germ was the object of a short confirmatory examination in the Hygienic Institute of Berlin, in the year 1888, by v. Esmarch, and in this it could be determined that the same answered, in the main, with Salmon's statements, as also that this bacterium corresponded with no cause of infection in pigs known (in Europe, D. E. S.) at that time."

How does it happen that unequivocal statements like this, on points of prime importance, should have been passed over in silence by the Committee on Intelligence and Education, while every effort was made to give the impression that the Bureau reports had been shown to be unreliable?

At the meeting of this Association in Baltimore in 1888, I read an extract from a letter received by me from Koch, in which was given the results of von Esmarch's examination of the culture of hog cholera germs which I had sent to Koch for his opinion. Billings quotes from that letter in his report, and his remarks on it show how completely wrong he was on the whole subject at that time. This is what he says:

"What does Koch's evidence show?

"Nothing, but that some unknown germ was sent to him that had some kind of effect on 'mice and guinea pigs,' but not that it had any on hogs. It does show that it does not occur in any swine disease known to him as occurring in Europe, and all the evidence quoted by me goes to show that the so-called swine plagues there are one and the same with that in this country, except the Wildseuche and Rouget." (l. c., p. 246.)

Again, he writes in the same connection:

"Has Mr. Salmon given a particle of evidence showing that this germ produces a single lesion, or does a single thing that that of swine plague does not?

"Then, reader, what is it?

"I will answer for you: An imposition!

"Not content with giving such condemnatory evidences against himseif, Mr. Salmon has gone so far as to pile it up so high that his eventual crushing into a worse specimen of nonentity than his 'hog cholera' psychosis is but a question of time. He has done this by sending a lot of cultures of his 'nonentity' to European investigators, all of whom say that they have never met with it, and that it does not occur in any case of swine plague known to them in their respective countries. They certainly 'settle the question' that there is no such pathogenic germ known to them, and I think the question has also been sufficiently settled that the real swine plague, with its one ovoid, belted germ, is either known to them all, or that there is evidence that it exists in their respective countries, except the, at present, somewhat peculiar character of the testimony from Sweden and Denmark with regard to the micro-organism, of which I have no fears that the little inconsistencies will eventually be cleared up."

The time has come when all of these little inconsistencies have been cleared up, but not in the way that Billings anticipated. After reading that von Esmarch found the germ to answer our description, and that Frosch finds that description so complete that no doubt can arise concerning its identity, and that this germ is the cause of the American swine disease, what can be our opinion of Billings' contention? And above all, what are we to think of our Committee on Intelligence and Education who demand that when the statements of the Bureau conflict with the results of men like Billings, they must be confirmed by independent investigators before any dependence is placed upon them?

I have not space to go into details concerning the many points of resemblance found in the description of the hog cholera germ given by Frosch, and in the description contained in the Bureau reports for 1885 and 1886. I will, however, enumerate the principal ones. The form and dimensions are given as exactly the same 1.2 to 1.5 u long by .6 u in breadth, both stating that they are liable to variation under different conditions. Both state that they are found in the blood and tissues, oftener in pairs than single; that while they stain readily by the usual solutions of aniline substances, the staining by Gram's method is unsuccessful; that they are actively motile in fresh cultures; that they grow upon the acid surface of the potato; that they do not liquefy gelatine, and that the gelatine colonies are brownish disks, often having a dark centre which makes them resemble a nucleated cell; that they grow upon the usual culture media in both room and incubator temperatures, presenting in each case the same appearance; that the time between inoculation and death is longer than with other septicæmic diseases; that in small animals it causes but slight local reaction; that mice, rabbits and guinea pigs were very susceptible, while pigeons require a dose of about 1 cc. to produce fatal results, and chickens are insusceptible; that in pigeons the pectoral muscle on the side of the inoculation assumes a parboiled or cooked appearance, while in inoculated mice and rabbits the liver becomes enlarged and presents numerous foci of coagulation necrosis.

In addition to the above, each author has mentioned some points which he considered of value, but taking simply the points which harmonize, we have a picture which clearly differentiates this germ from any other known micro-organism and which is amply sufficient for its identification. Frosch himself says, after giving the results of his examination of the culture sent by Billings: "If one now compares these results with the description of the Salmon hog cholera bacterium, then such a similarity of the two bacteria in the main points of morphology and biology is found, that their identity cannot be doubted, and it would be superfluous to again enter more closely upon the individual points in which they correspond."

If Frosch is correct in this opinion, and no one who has examined the question can have a reasonable doubt, and if Billings had in 1888 the same germ which he sent to Koch in 1889, what can be his justification for denying the existence of the hog cholera germ described in the Bureau reports? Upon this supposition, does not his own report make him out a bungler as an investigator, and an ignoramus in bacteriology?

Billings is now willing to have it assumed that he was working with the hog cholera germ from 1886 to 1888, and by so doing puts himself in a position where such unenviable charges naturally suggest themselves in connection with his work, because he finds even this embarrassment preferable to the other horn of the dilemma. I do not see how it can be admitted that the germ which he then described was the hog cholera germ.

If his plate on page 104 of his report, and fig. 3, plate 3 at the end of that work are examined, it will be evident that they do not represent the hog cholera germ, but rather that of the German swine plague. He insists in the text that this is the case, for he says: "We have thus described the normal, or general, cycle of development of the micro-etiological organism of the swine plague, the "*Wildseuche*" and hen cholera, as well as rabbit septicæmia, Texas fever and yellow fever, all of which diseases are caused by a member of this class of belted germs and should be classed as extra-organismal, local or land septicæmia." (l. c. p. 113.)

Again he says: "This concludes my observations of the micro-morpho-biological phases presented by these micro-etiological organisms in the course of their development." (l. c. p. 114).

Without stopping to discuss the nature of the micro-organisms of Texas fever and yellow fever, it may now be asserted, even on the authority of Frosch, that if Billings was describing the micro-morpho-biological phases of the germs of *Wildseuche*, hen cholera and rabbit septicæmia, he was not describing those of the hog cholera germ, because the latter is an entirely different micro-organism.

It surely is not necessary for me to quote extensively to prove that throughout Billings' report he repeats the statement again and again that the germ of his swine plague was morphologically identical with the germ of *Schweineseuche.* The idea runs parallel with, and is equally prominent with his other conclusion that no such germ as the hog cholera bacterium could be found in the American swine plague. In one place he says: "I emphatically declare that all Salmon's assertions in regard to the specific microscopical appearances of any true and specific micro-organism in connection with hog cholera to be one continued series of 'unfounded statements' down to the issue of his report of 1886." (l. c. p. 68).

And again, "It matters nothing to the hog raisers of the United States whether this germ was discovered by me on July 7, 1886, or by Salmon on July 1, or even in 1884." (l. c. p. 69).

Another remark is even plainer, as follows: "Can anyone comprehend what could have induced Mr. Salmon to say (of the true swine plague germ, but which he falsely asserts to be the cause of a 'chronic pneumonia," Report 1886) it is probably identical with the micrococcus described in my report of 1884," etc. (l. c. p. 392).

We see by the quotations which have been made not only that he considered the germ which he then had as identical with the *Schweineseuche* germ, but that he accepted the descriptions of the Bureau swine plague germ as applying to the germ described in his report. While he held that there was but one swine plague in the United States, he insisted that this was caused by an organism morphologically identical with the micro-organisms of *Schweineseuche*, hen cholera, rabbit septicaemia and the Bureau swine plague (see also l. c. pp. 293 and 332), and he denied the existence of a disease produced by the hog cholera germ.

Now, what was the effect of Frosch's report upon this position? Billings and the Committee on Intelligence and Education claim it as an endorsement, and if we accept it as such the query suggests itself, How many such endorsements can a writer have and survive? Billings and Frosch both claim that there is but one swine disease in this country which should be called a plague—the former says it is caused by the *Schweineseuche* germ; the latter says it is caused by the hog cholera germ. Billings says there is no disease produced by the hog cholera germ in the United States; Frosch says there is none caused by the swine plague germ. This certainly is one of the most remarkable endorsements on record.

With this condition of affairs before us we must inquire whether it is not possible that Billings had the hog cholera germ in his cultures previous to 1889 as well as after that date. Do not these germs resemble each other so closely that a bacteriologist might be excused for thinking he had one when he really had the other? So far from this being the case, these two germs are radically different, and any bacteriologist who confessed his inability to distinguish between them after studying them for two years, would certainly lose his reputation.

Frosch finds no difficulty in pointing out the most radical differences, although he evidently tries to let his friend Billings down as easily as possible. He shows that the hog cholera bacterium is motile and bears flagella, while the swine plague bacterium has no power of movement and no flagella; the former grows at the extremes of temperature of 8 degrees C. and 42 degrees C., the latter has no growth at either of these extremes, the growth of the latter upon gelatine and blood serum is much finer, more delicate and more clinging; the former in liquid cultures forms in twenty-four hours an equal turbidity and later a sediment, while with the latter there is only a slow growth found upon the bottom of the tube, always in flakes adhering to each other; in gelatine the colonies along the needle track with the swine plague bacterium are much finer and the surface growth more delicate, and on plates the colonies do not reach half the size of the hog cholera; the swine plague only grew on potato when the latter was alkaline, and as it is usual for boiled potatoes to have an acid reaction, the germ would not grow upon them, while the hog cholera grew abundantly when they were acid; the hog cholera growth on potato was always luxuriant and of a dirty light-yellow color, that of swine plague (on alkaline potato) formed flat, not very extensive, gray or yellowish gray fields. The hog cholera grows better without oxygen than the swine plague, and in general the former has a greater growth-energy. Agar tubes colored with lacmoid and sulph-indigotate of soda were discolored by the hog cholera germ, while those with litmus remained unchanged; with the swine plague the litmus and indigo tubes were unchanged, but the lacmoid was slightly discolored. The swine plague germs produce phenol and indol during their growth, the hog cholera, neither of these bodies; while the size is variable, the latter in general produces coarser forms.

Guinea pigs have a certain power of resistance to the swine plague, but are exceedingly susceptible to the hog cholera; the reverse in true concerning the susceptibility of pigeons. In all susceptible animals death occurs from two to three days sooner after inoculation with swine plague than with hog cholera. The local reaction is as insignificant and trifling for the hog cholera germ as it is remarkable and severe for the swine plague. The hog cholera produces multiple, coagulation necrosis in the acini of the liver, while when inoculated with swine plague this organ tends to undergo fatty metamorphosis. Both agree in that they are found in the blood, that they are most numerous in the spleen, and next to this in the glandular, parenchymatous organs, as also in the muscles of the body. They differ in that the blood of the ventricles and auricles, as also that of the main blood vessels is comparatively poor in hog cholera bacteria, while in acute cases of swine plague these localities are just as rich in bacteria as the above-named organs. In hog cholera some of the capillaries are found fully plugged with rod-like bacteria, while in the region lying between, no bacteria can be observed; the swine plague bacteria on the contrary are evenly distributed in the capillaries and are never so thick as to cause plugging. Finally the swine plague germ is found in great numbers, almost in pure cultures, in the inflammatory œdema at the seat of inoculation, as also in the not infrequent exudates in the thoracic and abdominal cavities, while the hog cholera germ is so sparingly present at the seat of inoculation, as well in the small, hardly more than normal amount of fluid in these body cavities, that it can often be detected only with the plate culture method. To these differences enumerated by Frosch, we may add that the hog cholera germ is most frequently seen in pairs when cover-glass preparations from the organs of the affected animal are made, while the swine plague germ under the same conditions is generally single.

I have summarized these points of difference from Frosch's paper in order to ask if it is possible for any bacteriologist to mistake one of these germs for the other after he has studied them in cultures and by inoculating animals with them? And yet to assume such a mistake is the only way in which we can harmonize the germs sent by Billings to Koch with the description in the former's reports.

I am aware that some of the statements in his reports indicate that the hog cholera germ was under investigation, while others would indicate that it was the swine plague. For instance, the statement that he watched the development of the germs in a hanging drop culture would be absurd applied to the actively-motile hog cholera germ. Frosch tried to follow him in this, but gave it up as impossible. On the other hands the luxuriant growths on potatoes which he described could never come from the swine plague germ. Again the first cultures which he sent to Europe are said by Bunzl-Federn to have developed acidity in milk cultures and to have formed phenol and indol, which shows them not to have been the hog cholera germ, while later he undoubtedly sent the hog cholera germ to different investigators. If we accept these various statements of facts we can only conclude that he at one time had one germ and at other times the other germ; indeed, this is indicated by his inoculation experiments in which some of the animals died too soon for death to be caused by the hog cholera germ, and others after too long a period to indicate the swine plague germ.

It is no task of mine, however, to explain his inconsistencies and contradictions. I should not have taken the trouble to point them out, had his work not been held up to this Association in connection with Frosch's report in the attempt to show the reports of the Bureau of Animal Industry to be unreliable. I have shown you how Billings' conclusions stand, or rather fail to stand, when tried by Frosch's investigations. Now let us see how the Bureau's conclusions fare when compared with these and other recent investigations.

In 1885 the Bureau described the germ of hog cholera—a germ so different from any previously described in swine diseases, that for a long time our conclusions were not accepted as correct by any other investigators. In 1886, soon after Schütz's report appeared, we showed that the germ of hog cholera was essentially different from that of the German *Schweineseuche*, and that we had another disease with a germ which appeared to be identical with the germ of *Schweineseuche*. It is unnecessary at this time to go into particulars as to how these conclusions have been contested, both at home and abroad; but fortunately they are now both vindicated by independent investigators.

As to the hog cholera germ, its pathogenic character and its difference from the *Schweineseuche* germ, we need no further evidence than is given by Frosch. The latter, however, has seen fit to contest the specific and pathogenic character of our swine plague germ, particularly as related to a plague of equal distribution with hog cholera. This opinion of his is not founded on any investigations of his own, but upon captious criticisms of our methods. It may be said in the first place, that we never claimed that swine plague had an equal distribution with hog cholera. This was a question which could only be decided by more extensive investigations than we have been able to make. We did say that it was a widespread plague, and we see no reason to change our views.

As to whether Frosch was right in his assumption that the swine plague germ discovered by us was not the cause of a specific disease, distinct from hog cholera, we must decide by the results obtained by other investigators. The Commission of Inquiry recognized the existence of such a disease; Welch, of Johns Hopkins University, has studied it; Jeffries had undoubtedly cases of it; and even Billings, after first accepting it as identical with his swine plague and later denying its existence, comes around finally and admits that he has seen a few cases.

One of the most satisfactory studies of the question is that made by Afanassieff at the request of Baumgarten. It will be remembered that the latter, judging from Billings' reports, had stated his belief that there was but one swine plague to be considered as existing in this country, and that this was identical with the German *Schweineseuche*. The result of the studies of Afanassieff is a complete confirmation of the reports of the Bureau. Not only does he confirm Frosch in showing that the Billings swine plague germ as at present sent out is identical with our hog cholera germ, and different from the *Schweineseuche*, but he finds that our swine plague germ is practically identical with the *Schweineseuche*, as we concluded six years ago.

The whole subject of the characteristics and pathogenic effects of the hog cholera and swine plague germs may now be regarded as cleared up by the investigations of independent workers in Europe. For years Billings, supported by others who should have known better, succeeded in keeping up doubts as to the facts, and particularly as to the correctness of the Bureau reports. For our part we have patiently waited until independent workers should take up the subject and settle it by laboratory researches, instead of by polemical writings. We have had a long time to wait, but our work is now confirmed on all sides, and our Nebraska antagonist is left between the two horns of a dilemma, either of which is fatal to his pretensions as a bacteriologist.

So much for the fundamental questions involved. But since our committee tells us that "work which is recognized as valuable abroad cannot be ignored at home," it will be interesting to briefly examine some of the collateral issues which have been raised. In his report, from which I have already quoted, Billings writes :

"The interesting question now is, What kind of an organism is Mr. Salmon sending round as the 'specific microbe of hog cholera'?

"It shows a singular want of scientific exactness that Rietsch, of Marseilles, who had it while making his own investigations, does not give any description of this thing. It has been absolutely impossible for me to get any culture of this organism, but last summer Dr. Detmers sent me a slide marked as follows: 'Bacterium Salmonidi,' on one label, and on the other, 'Doctor B. Persh, culture in gelatine, 10, 24, '86.' This organism bears no relation to anything described by Mr. Salmon, either as to the germ of swine plague in '85, or of his hog cholera in '86, except perhaps in outline morphology ; it is a small, ovoid organism, about three times as long as wide, and has rounded ends, but colors, or is colored, entirely, and it does not seem as if the tinction had been too strongly applied. Sometimes, when in process of fission, there does seem to be a very slight, clear space in the middle of the body, but in no way does it resemble Mr. Salmon's description or plates, with the exception of the very latest, where the ends do color somewhat.

"Is this Mr. Salmon's 'hog cholera' bacterium ?

"As to that I know not, but I will permit Dr. Detmers to tell his own story about it."

This is very peculiar writing for a bacteriologist. The organism he says bears no relation to anything described by me except perhaps in outline morphology. In what other respect would he expect a resemblance in a mounted preparation of germs ? We certainly have had very little information from any writers as to the internal structure of bacteria. What he means, no doubt, is that the staining did not correspond with the description in the Bureau reports. It should be remembered, however, that that description was expressly stated to apply to the germs as stained in cover-glass preparations from the blood and organs of affected animals. Our photographs of stained germs from cultures, given in the report for 1886, shows the germs solidly stained.

It is certainly well known to every bacteriologist that germs of the same species, grown in different media, vary morphologically and, therefore, if a description is to be verified the conditions must be the same. The typical preparation showing the end-staining of the swine plague germs and the unstained middle belt are most reliably obtained from the blood and other tissues of affected animals. To make a comparison the hog cholera germs should be obtained from the same source.

The first thing that strikes one in staining such preparations is that the swine plague germ still shows its peculiar staining perfectly when kept in the coloring liquid a length of time that would make the hog cholera germ a uniform color throughout. Consequently, in staining the latter germ more discretion must be used to obtain its typical appearance. The typical appearance is much more difficult to obtain with the organism which has been grown in various culture media, as the tendency to produce a uniform color is much stronger. If, therefore, the germs sent to Billings by Detmers were colored entirely, that is the proper and only possible indication that the "tinction" had been too strongly applied. He goes so far as to admit, however, that "sometimes there does seem to be a very slight, clear space in the middle of the body," but he is careful to add that "in no way does it resemble Mr. Salmon's description or plates." It would have been interesting if he had pointed out how a clear space could exist in the middle of a germ and still not resemble in any way another clear space located in the same position in another germ.

These inconsistencies show that the whole argument lacks candor or even a desire to reach the truth.

It may be freely admitted with Frosch and, as we have shown in our reports, that this germ, in common with many others, varies greatly, even in its staining properties, when obtained from different outbreaks or when grown under different conditions. The same is true of the swine plague germ. This, however, does not prevent each from having its peculiar staining, which is valuable as a diagnostic point, when taken in connection with other morphological and biological characters, but which alone is not sufficient to determine a species.

That the figures of the hog cholera germ in our reports do vary somewhat as to the staining, as charged by Frosch, is not denied, but he surely is the last one to consider this as bearing against the value of our statements, for he expressly calls attention to the variations which are liable to occur and to the delicacy of the manipulations necessary to get comparative results. Our figures were drawn from the natural objects, and were correctly drawn; if they had been diagramatic plates, such as adorn Billings' report, then an exact correspondence might have been expected. In spite of the variations, however, the difference in staining between the hog cholera and swine plague germs is very apparent in our plates, and can be relied upon as exact.

With organisms which vary in many of their characters to the extent which occurs in bacteria from slight changes in the conditions of life, it goes without saying that it requires skill to secure typical preparations, and even then such are not to be expected from all outbreaks. For that reason a safe diagnosis cannot be made from a single character, and above all is it unsafe and unscientific to reach positive conclusions from the simple microscopical inspection of a preparation, the germs of which have been cultivated, stained and mounted by another person.

The examples of Billings' recklessness in this respect, and of his discomfiture, are numerous and instructive. Dr. Shakespeare, previous to his appointment on the Commission of Inquiry, sent him a photograph of a certain micro-organism, and inquired if it resembled the germ found by him in the swine plague of Nebraska. Billings at once accepted it as identical with his swine plague germ, showed it among his friends, and wrote it up in the newspapers as another proof of the correctness of his own conclusions and the incompetency of the Bureau. When the commission reached Lincoln, the members were invited to meet a number of scientists and people connected with the University, and at this gathering Billings made an address. Among other things, he made a strong point of the confirmation which his work had received from the investigations of Shakespeare, and exhibited the photograph as proof positive that the latter had isolated the same germ from hog cholera which he himself claimed to be the cause of that disease. To no one was this greater news or more of a surprise than to Shakespeare himself, who was obliged in self defense to explain that the germ in question was not obtained from a sick hog, but from a person affected with some bowel disorder, and he had no reason to suppose it had any relation to any swine disease.

His alleged discovery of the germ of yellow fever is only another example of the same ridiculous blundering. He claimed to have found the organism discovered by Babes, in 1885, in specimens from seven cases of yellow fever, and that in only one case was there any pollution. Of these investigations he wrote: "I am willing to risk some considerable reputation that the germ herein described as present in all this material is the specific cause of yellow fever, as well as that the description of the morpho-biological phenomena presented by the germ of the Southern cattle plague will largely be found applicable to this." (Original Investigations of Cattle Diseases in Nebraska, 1886–89, p. 116.)

Now, what are the facts about this? In his recent report Sternberg gives a history of this material and of the results of a careful examination of it by different experts. The bacillus of Babes, alleged by Billings to be present "in every section and in great numbers," is conclusively shown to have been absent from the specimens which he referred to with greatest confidence, and probably did not exist in any of the specimens which he examined. These specimens did contain the *bacterium coli commune* and other organisms which represented a *post mortem* invasion of the tissue. (Report on the Etiology and Prevention of Yellow Fever, by George M. Sternberg, Washington, 1890, pp. 176–180.) His identification of the Babes germ is therefore on a par with his identification of his swine plague germ in the photograph sent him by Shakespeare, and his much vaunted yellow fever investigations turn out to be a farce of the most absurd character.

To come back to Billings' opinion of the slide sent him by Detmers, it can be seen that it was formed with the same kind of evidence as his opinion of the Shakespeare photograph and of the yellow fever germs. As evidence of the kind of germ, this opinion, from the nature of the case, can have no weight, but as evidence of the care, or lack of care, shown in reaching his conclusions, it is incontestable.

Detmers, who studied the germ from which this slide was made, both in cultures and by the inoculation of a rabbit, decided that it was entirely different from his swine plague, and, as Frosch remarks, " on the very remarkable ground that the *post mortem* lesions found in the rabbit were not like those he was accustomed to see in pigs." Detmers and Billings were both positive on two points; first, that their swine plagues were identical, and, second, that this germ discovered by the Bureau was entirely unlike the germ of their disease.

Detmers said: "I cannot identify it with swine plague. If it is identical with it, then I must say the disease I heretofore pronounced swine plague, or, as the farmers call it, hog cholera, must be bogus; that is, something else. I am compelled to pronounce the disease of which the rabbit died a fatal septic disease entirely different from swine plague." Again, he says of the disease produced by this germ: "It is a septic germ; readily kills rabbits and causes septicæmia, but has no connection with the disease in question. It is not for me to say where Dr. Salmon obtained it, or from where he may have imported it." (Billings' Report on Swine Plague, pp. 249 and 257.)

Billings himself took the same ground, for he remarked: "Swine plague itself being a septicæmia, may it not be possible that Mr. Salmon has accidentally dropped upon some kind of a septic organism capable of producing the same lesions?

"This question has been frequently in my mind of late, for if not so, I do not know how to answer for all Mr. Salmon's wonderfully positive experiments. If not so, then these experiments were never made, and the whole thing is a concocted farce, or else he has been secretly using the real germ and showing people this other thing. I am determined to force this question to such an issue that the real facts must come out sooner or later, for I, as repeatedly said, have no fears but what my own work will stand every test." (l. c. p. 249).

Here are the two positions; which side was correct? The investigations of von Esmarch, Frosch, Bunzl-Federn, Raccuglia, Afanassieff and others leave no doubt as to the reality and pathogenic effect of our hog cholera germ. Billings himself has since adopted it, but what microbe did he and Detmers have at the time they were making such charges against the Bureau? Surely, if they had ever seen the hog cholera germ they would have recognized it in this culture which Detmers obtained from Persh. If they had not seen it, then what becomes of the claim made by them both that Detmers is entitled to the credit of its original discovery?

I do not propose at this time to follow these critics through any more of their inconsistent and contradictory writings. I have done this much to show you the quality of the investigations which are held up to you because they are "recognized as good abroad and cannot be ignored at home." If the general conclusions of a man's investigations are so absolutely wrong, how can the details upon which these are founded be any nearer right? We might and should excuse errors, or apparent errors of detail, but how can you accept a work which is wrong in its principal conclusions?

The principal conclusions of the Bureau reports as to the existence of the hog cholera and swine plague germs in this country have been confirmed on all sides. The doubts of European investigators as to the correctness of these conclusions have been withdrawn. What discoveries of value have been made regarding the biology of the hog cholera or swine plague germs by other American investigators and have been satisfactorily confirmed? If on the other hand you compare the report of Frosch with the Bureau reports, you cannot fail to observe how many of his conclusions were previously announced in our reports.

Does this Association take no pride in the investigations of the Bureau of Animal Industry, or does it propose to stigmatize them as a disgrace to the veterinary profession? Is it willing to brand them as discreditable, and the men connected with them as ignorant, incompetent and dishonest, on the evidence which has been submitted? Do you forget that Billings has vilified about every scientist whose reputation might serve him as a stepping stone if he could once get it under his feet?

Why, then, are these reports of his which contain no discoveries of value, if they contain any discoveries at all, reports which are filled with the most brutal personalities, which give no account of experiments conducted in a scientific manner, which are lacking in those details necessary for their confirmation by other investigators, which above all are manifestly wrong in their principal conclusions —why are these reports now held up before us as too good to be ignored and the reports of the Bureau of Animal Industry, written in part by members of this Association, denounced as they have been? Are you aware that this report of our committee was no sooner published than it was circulated broadcast by Billings as another vindication of his work and his methods? Do you not know that he is repeating with Texas fever the role which he played with swine diseases—denying the existence of the true germ and claiming the discovery of something else? Why should this Association go out of its way to assist such a man in obstructing the progress of science and in vilifying its own members?

Gentlemen, I leave the matter with you, knowing that my own work has been carefully and conscientiously performed, and with the full confidence that every detail in the work of my scientific assistants has been done with equal care and honesty. It is for you to decide whether such reports from one of our leading committees are to be endorsed and encouraged. I express no opinion as to the honesty of the committee, but I do charge that its report was incorrect in both of its statements and its conclusions, and inexcusable in its personalities. I do insist that this Association cannot afford to endorse such a report or to allow others like it to be presented in the future, whether they affect me or any other member of this Association. The truth will finally prevail, no matter what our action may be; but if such mischievous reports continue to come from a leading committee, you will be responsible for the loss of prestige, and influence to the Association and the loss of good will between members, which must necessarily follow.

